# Psychological talent predictors in youth soccer: A systematic review of the prognostic relevance of psychomotor, perceptual-cognitive and personality-related factors

**DOI:** 10.1371/journal.pone.0205337

**Published:** 2018-10-15

**Authors:** Dennis Murr, Philip Feichtinger, Paul Larkin, Donna O‘Connor, Oliver Höner

**Affiliations:** 1 Institute of Sports Science, Eberhard Karls University, Tübingen, Germany; 2 Institute for Health and Sport, Victoria University, Melbourne, Australia; 3 Sydney School of Education and Social Work, The University of Sydney, Sydney, Australia; University of Lleida, SPAIN

## Abstract

Within the multidimensional nature of soccer talent, recently there has been an increasing interest in psychological characteristics. The aim of this present research was to systematically review the predictive value of psychological talent predictors and provide better comprehension of the researchers’ methodological approaches and the empirical evidence for individual factors (i.e., psychomotor, perceptual-cognitive and personality-related). Results highlighted heterogeneous study designs (e.g., participants, measurement methods, statistical analyses) which may limit the comparability of studies’ findings. Analyzing the number of included studies, psychomotor (*n* = 10) and personality-related factors (*n* = 8) received more consideration within the literature than perceptual-cognitive factors (*n* = 4). In regard to empirical evidence, dribbling (0.47 ≤ *d ≤* 1.24), ball control (0.57 ≤ *d* ≤ 1.28) and decision-making *(d* = 0.81) demonstrated good predictive values as well as the achievement motives hope for success (0.27 ≤ *d* ≤ 0.74) and fear of failure (0.21 ≤ *d* ≤ 0.30). In conclusion, there is growing acceptance of the need for more complex statistical analyses to predict future superior performance based on measures of current talent. New research addresses the necessity for large-scale studies that employ multidisciplinary test batteries to assess youth athletes at different age groups prospectively.

## Introduction

Talent identification, selection and development of youth soccer players is an important issue for clubs and soccer federations, as they are challenged to find talented youth players who may have successful professional careers in adulthood [1]. Thus, talent development programs aim to detect players’ potential for future success at a young age. However, with the multifaceted characteristics of sport performance and the high inter-individual differences during athlete development [2], this endeavor remains highly challenging [3]. Due to the complex nature of the developmental process from youth player to elite status, which depends on various interacting personal and external factors, a multivariate and dynamic approach to research is required [4, 5].

In order to understand the intricacy of these predictors, a multidimensional spectrum of potentially prognostic relevant factors has to be considered [5]. This is acknowledged by Williams and Reilly [6], who developed a heuristic model for the categorization of talent predictors, which identifies potential predictors in four sport science dimensions, including, physical, physiological, psychological and sociological characteristics. Within this multidimensional spectrum, both in research and practice, there has been increasing interest in the psychological dimension [3]. More specifically, the psychological area comprises psychomotor, perceptual-cognitive and personality-related factors [7].

Usually, players are evaluated by experienced coaches or scouts who make subjective judgements on their potential based on current levels of performance [8]. In order to provide a more comprehensive understanding of the identification and selection process, there has been growing support for more scientific evidence assessing relevant talent predictors with objective diagnostics that may indicate future sporting success [9].

Wilson et al. [10] demanded a stronger consideration of psychomotor factors for talent research and acknowledged that players with good *psychomotor factors (e*.*g*., *technical skills)* are highly coveted players. This is supported by current findings which highlighted techniques such as dribbling and ball control are the most frequently performed skills during a soccer match [6]. Several authors (e.g., [5, 11]) reported evidence to support the notion of testing technical skills as a discriminating factor between playing levels in youth soccer. For example, dribbling performance was acknowledged as an important indicator when comparing the performance of elite and sub-elite youth players [5]. Höner, Leyhr, and Kelava [12] reported higher predictive power for the latent factor variable “technical skill” (consisting of dribbling, ball control and shooting) compared to “speed abilities”. In a professional team context, Rampinini, Impellizzeri, Castagna, Coutts, and Wisløff [13] established that during matches successful teams (ranked in the first five positions of the Italian Serie A league) completed a higher passing rate as well as more dribbling actions and shots on target compared to less successful teams (ranked in the last five positions).

*Perceptual-cognitive performance factors* such as anticipation and decision-making have been found to be crucial for soccer players [14]. More specifically, the ability to anticipate what is likely to happen in the next situation is as important for soccer performance as the ability to decide and execute suitable actions in certain situations under time constraints. Several studies proved the importance of these factors with regard to discriminating players in performance level, age group or playing position [15–19]. For example, Kannekens, Elferink-Gemser, and Visscher [20] recently highlighted that besides technical factors, tactical facets (e.g., decision-making) are critical when identifying talented youth soccer players.

Regarding *personality-related factors*, talent models (e.g., [6, 21, 22]) consider psychological dispositions (i.e., the tendency to…) and mental skills (i.e., the ability to…), predominantly within the areas of motivation, volition, self-referential cognition, and emotion. The research on motivational characteristics and their relationships with performance in soccer (e.g., [23–25]) has predominantly focused on achievement motives (i.e., dispositions that provide information about how individuals perceive and evaluate achievement situations [26]) and motivational orientations of athletes (i.e., dispositions that provide information about the criteria that individuals use to define success and judge their level of ability [27]). Further research has addressed how volitional competencies are associated with performance in soccer and has focused principally on aspects of self-regulation such as reflection and effort [28]. Regarding self-referential cognition, physical self-concept (i.e., the aspects of general self-concept that comprise any self-referential information about a person's own body [29]) and self-efficacy (i.e., a person's belief in his or her own capabilities to succeed in specific situations [30]) can be regarded as particularly relevant. Previous studies in soccer have also considered self-confidence to be relevant for performance (e.g., [31]). With respect to emotional characteristics, research has focused on competition anxiety as an important factor that can influence soccer performance [31, 32].

Regarding the current state of research, several studies have revealed the importance of psychological factors in soccer (e.g., [33]). While the majority of these studies are cross-sectional in nature (i.e., comparing performance between known age groups or performance levels [23, 34, 35], more recently, researchers have attempted to use more longitudinal study designs to assess the stability and/or predictive value of psychological factors for future success [36]. At this stage, however, there exists no systematic overview of such studies. Previous reviews provide important knowledge by analyzing the impact of personality traits on perceptual-cognitive skills [37] or reviewing the relevance of psychosocial factors associated with talent development [38]. While Johnston, Wattie, Schorer, and Baker [36] systematically reviewed the efficacy of talent identification programs in predicting levels of achievement in sports generally, researchers have yet to systematically review existing empirical studies with regard to the prognostic relevance of psychological talent predictors in soccer. To analyze the value of the prognostic relevance it is important to consider the mostly inhomogeneous study designs of the individual studies. It is therefore central to take into account design features (e.g., participants, measurement methods, design and statistical analysis) that may influence the predictive value.

In 2000, Williams and Reilly [6] provided a narrative review of prognostic studies in soccer and suggested a heuristic model in which personal talent factors were propagated as physical, physiological and psychological predictors. With respect to physical and physiological talent predictors in soccer, Murr, Raabe, and Höner [39] highlighted in their systematic review the prognostic value of these “non-psychological” predictors. The present systematic review extends this knowledge leading to a comprehensive overview about prognostic relevance of personal talent predictors. Therefore, this systematic review focusses on psychological talent predictors and aims to improve the understanding of the current research via two objectives. *First*, existing research exploring the prognostic value of psychological factors for youth soccer players was systematically reviewed. Furthermore, relevant design features (i.e., methodological issues) of the included studies were examined in order to provide an overview of the researchers’ methodological approaches (objective 1). *Second*, the empirical evidence for the individual predictors found in the reviewed studies was described precisely (objective 2).

## Method

The systematic review was conducted in accordance to the Preferred Reporting Items for Systematic Reviews and Meta-Analyses (PRISMA) guidelines [40]. Given these guidelines were originally developed for clinical studies, some items of the PRISMA checklist (S1 Table) could not be reported as they are not relevant for the current systematic review.

### Procedures

This current study on psychological predictors complements the systematic review of the prognostic relevance of physiological and physical characteristics in soccer by Murr et al., [39], having the same initial procedures in common: Studies were included on the basis of the following criteria (i) study sample consisted of youth soccer players (≤ U19); (ii) studies predicted future success based on fitness, anthropometric, and/or psychological diagnostics; (iii) studies included an assessment of physiological, physical, and/or psychological characteristics; (v) information about participants’ future performance was provided; (vi) statistical indices for prognostic relevance are given; and (vii) articles were peer-reviewed published in English or German (the authors native language) between 2000–2016. Afterwards, studies in the overarching project were excluded if they did not investigate psychological predictors.

### Search strategies

To identify potentially relevant articles from applicable databases (i.e., Academic Search Premier, Medline, PsycArticles, Psycinfo, PsycTESTS, PSYINDEX, PubMed, SPORTDiscus, Web of Science Core Collection) the following combination of search terms (in both English and German) was used:

$$\begin{array}{c} \left[Soccer\;OR\;football\right]\;AND\;\left[Youth\;OR\;elite\;OR\;talent\;OR\;junior*\;OR\;adolescent*\right]\;AND\\ \left[Diagnos*\;OR\;test*\;OR\;predict*\;OR\;prognos*\;OR\;identif*\;OR\;select*\;OR\;develop*\right]. \end{array}$$

A final electronic search for each database (time span for searches: 1 January 2000 to 31 December 2016) were performed on January 5th, 2017. The initial search identified 13,320 relevant articles across all databases. After removing duplicates—both manually and automatically (using Endnote X7) - 7,800 articles remained.

### Article screening

**Fig 1:**
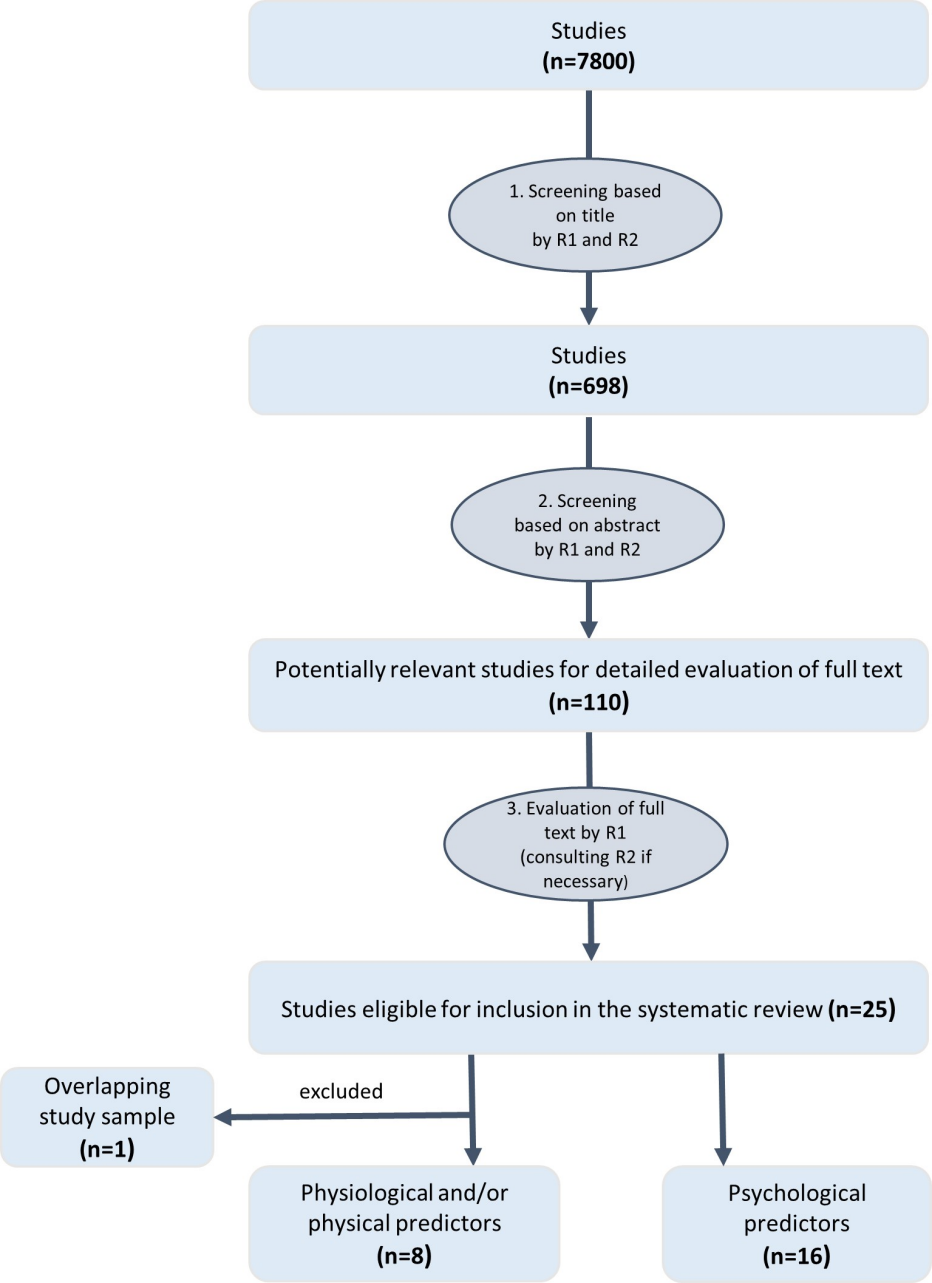
Flow diagram of the selection procedure.

Two reviewers (i.e., the first author and a research assistant) screened the articles independently to find relevant studies that met the defined inclusion criteria. The selection process consisted of four stages (see Fig 1). In the *first* stage, both reviewers screened article titles against the inclusion criteria. In total, 698 articles were retained for review, with a 93.86% agreement between the reviewers (articles were retained, if at least one of the reviewers argued for inclusion, otherwise, they were excluded). *Second*, the remaining 698 abstracts were evaluated against the inclusion criteria by both reviewers with 110 articles retained (90.11% agreement between the reviewers). In the *third* stage, the first author reviewed the full texts against the inclusion criteria. Any uncertainty about the appropriateness of an article was resolved through a discussion and consensus approach by the first author and a research assistant. *Finally*, 16 studies were deemed to have satisfied all inclusion criteria relating to psychological predictors.

### Data extraction and statistical analysis

To address the first objective, a descriptive overview of existing research was prepared for three different psychological factors (i.e., psychomotor, perceptual-cognitive, personality-related). In this context, the following central features of the study were operationalized (see Table 1) and analyzed in their appearance. The **participants** were differentiated by *sex* (i.e., female or male) and *country* of origin where investigations took place. *Soccer development stages* were categorized based on established classifications of talent development programs (e.g., German Football Association’s talent identification and selection program [41]). The participants’ *performance level* at the time of the initial survey (measurement T1), was separated pragmatically based on its appearance in the included studies. In relation to studies’ **measurement methods** *diagnostics* were described listing the type of instrument administered to elicit predictors (e.g., self-report questionnaires, video-based tests). The operationalization of the *criterion variable* (participants’ performance at the time of subsequent measurement T2) followed (as best possible) the terminology utilized by the authors of the included studies. With respect to study **design and statistical analysis,** the number of players who participated in the studies was considered as statistical power to find the significance of the relationship between two groups linked to *sample size* [42]. Furthermore, the time interval between initial data collection (T1) and determination date of future success (T2) can have an impact on an individual factors’ predictive relevance. Therefore, it is important to consider different length *prognostic periods*. To address the complex characteristics of performance, the *dimension of domains* (i.e., number of psychological predictors’ domains that were investigated) were examined. In this context, it is worthwhile to compare procedures based on the distinction between different *statistical approaches* (e.g. ANOVA, MANOVA, LICUR). It should be noted that several authors investigated overlapping development stages or conducted studies with different prognostic periods. This led to several studies being reported in numerous catergories and consequently the results could indicate more than the 16 included studies.

**Table 1:** Overview of relevant design features of existing research on the prognostic value of psychological factors.

Participants		Measurement Methods		Design and Statistical Analysis	
*Gender*	female	*Diagnostic*	type of instrument	*Sample size*	N < 100
* *	male	Criterion variable* *(Selection level T2)*	PRO	* *	100 ≤ N ≤ 200
*Continent*	Europe		NT or YA	* *	N > 200
* *	Australia	* *	SCHO	*Prognostic period*	< 1 year
*Development stage*	<U12	* *	CR	* *	1–3 years
* *	U12-U15	* *	NEXT	* *	> 3 years
* *	U16-U19			*Dimension of domains*	unidimensional
*Performance level T1*	youth academy				multidimensional
* *	talent development program			*Procedures*	univariate
	regional clubs				multivariate
* *					person-oriented

Note.

*Criterion variable at the time of subsequent measurement, assessing whether individuals turned professional (PRO), joined a youth national team or a youth academy (NT or YA), received a scholarship for an elite program (SCHO), performance was rated by their coaches (CR) or simply reached next age class at the competitive playing level (NEXT).

To determine the *methodological quality* of the studies, an adapted version of the Critical Review Form for Quantitative Studies by Law et al., [43] was implemented. This modified assessment tool has been used in a systematic review of talent identification and development in soccer by Sarmento, Anguera, Pereira, and Araújo [44]. To assess the methodological quality of the studies in the present review, only criteria from Sarmento et al. (see p. 990 in [44]) that are related to studies’ methodological design features were applied: “appropriateness of the study design (item 3), sample included (items 4 and 5), informed consent procedure (item 6), outcome measures (item 7), validity of measures (item 8), method description (item 9), significance of results (item 10), analysis (item 11); see S2 Table). The ratings per quality criteria were 1 (meets the criteria), 0 (does not meet the criteria fully or is not described).

Next, the number of studies that investigated each psychological dimension and the frequencies of individual measured predictors (e.g., dribbling, decision-making, achievement motive) were reported. Furthermore, effect sizes and significant results were highlighted in order to analyze the empirical evidence for these individual predictors (objective 2). Cohen’s d were obtained from two-group comparisons reported in the individual studies with regard to the different development stages (<U12, U12-U15, U16-U19). In case Cohen’s d was not indicated in the original articles or only was investigated for individual age groups, reconstructed effect sizes for the development stages based on descriptive statistics were attained using the equation of Cohen’s d with the pooled standard deviation [45]. The effect sizes were characterized as small (0.2 *≤ d <* 0.5), moderate (0.5 ≤ *d* < 0.8), and large (*d* ≥ 0.8) based on the recommendations of Cohen. To determine significant group differences (*p <* 0.05) independent t-sample test were conducted. In accordance with Murr et al. [39], when descriptive results were not presented or a holistic approach (e.g., person-oriented approach where clusters are formed) was used (*n* = 5), the authors of these studies were contacted by the researchers and were asked to provide their descriptive statistics for effect size computation. Furthermore, a reconstructed Cohen’s d was also used when researchers compared three or more groups. Therefore, all analyses were restricted to a comparison of participants from the highest and the remaining competitive levels (i.e., “best” vs. “middle” and “weaker” players). In one study in which coaches rated the performance of their players, a product-moment correlation between coaches’ rating and individual predictors was computed by employing the Fisher Z-transformation.

## Results

### Objective 1: Analysis of relevant design features of existing research on the prognostic value of psychological factors

#### Participants

In general, all 16 reviewed studies investigated male youth soccer players, with no study examining the performance or characteristics of female youth soccer players. In terms of location, 15 of these studies were conducted in Europe (i.e., Austria, Belgium, Finland, Germany, Netherlands, Portugal, Spain and Switzerland) and one in Australia. With regard to developmental stage, the talent stage (U12-U15, *n* = 12) was the most frequently investigated, with the elite stage (U16-U19) examined in seven of the articles and the foundation stage (< U12) accounting for two of the studies. Relating to the participants’ performance level, a total of eight studies (50%) explored predictors of players attending a national youth development program, seven studies investigated prognostic validity with players from a youth academy, and one study examined predictors with regional club players [46].

#### Measurement methods

Concerning diagnostics, specific types of instruments were applied in the studies to investigate variables in the three domains of predictors. To assess technical skills, the majority of authors (*n* = 9) used soccer-specific motor tests, with six implementing tests developed by several soccer federations (i.e., Football Association of Finland, German Football Association, Portuguese Football Federation). Only Gravina et al. [47] and Huijgen, Elferink-Gemser, Lemmink, and Visscher [48] implemented non-soccer specific assessments. Of the four studies that measured perceptual-cognitive factors, three utilized the Tactical Skill Inventory for Sports (TACSIS; [49]), and one study used a video-based diagnostic. In the area of personality-related factors, all of the eight studies used self-report questionnaires. Each psychological disposition or skill was assessed by one particular measurement instrument, for example, the ‘Achievement Motive Scale’ (AMS) was always used to measure achievement motive. However, motivational orientations were addressed using two different questionnaires, either ‘Task and Ego Orientation in Sport Questionnaire’ (TEOSQ) or ‘Sport Orientation Questionnaire’ (SOQ).

The operationalization used by the researchers to describe the selection level at the time of subsequent measurements (T2) varied greatly. Four studies examined whether players reached the next age group at the same competitive level or achieved a professional status. Other authors used the selection for a youth national team (*n* = 5) or youth academy (*n* = 2), a coaches rating of player performance (*n* = 2), or obtainment of a scholarship for an elite program as criterion variable at T2 (*n* = 1).

#### Design and statistical analysis

The majority of the 16 studies consisted of sample sizes between 100 and 200 (*n* = 9). The remaining authors conducted their investigations with less than 100 (*n* = 3) or more than 200 (*n* = 4) participants, with the investigation by Höner and Votteler [50] consisting of a sample of 22,843 players. The prognostic period varied from less than one year (*n* = 5) to 15 years (i.e., [51]). The majority of the studies utilized middle term prognostic periods (1–3 years, *n* = 7). Investigations with prognostic periods longer than three years were performed in six studies. With regard to dimension, two studies [48, 52] examined talent predictors in all three psychological domains. Three authors conducted investigations of only two psychological factors. The majority of the studies were unidimensional divided between technical skills (*n* = 6), perceptual-cognitive factors (*n* = 2) and personality-related dispositions or mental skills (*n* = 3).

With respect to statistical analysis 62,5% (*n* = 10) of the studies conducted univariate analysis (e.g., ANOVA, two-sample t-tests, logistic regression), and the remaining six applied a multivariate method (e.g., MANOVA, structural equal modelling) linked with follow up analyses. Finally, in the research project of the Swiss talent promotion program [53] a holistic concept was applied using a person-oriented approach (based on LICUR method).

#### Methodological quality of the studies

For all eligible studies, the applied methodological quality criteria were almost fulfilled exclusively (see supplementary material 1). Ten of the 16 studies (62.5%) met all nine criteria (i.e., [1, 20, 48, 50, 54–59]), while five studies (31.3%) fulfilled eight of the criteria (i.e., [46, 51–53, 60]) and one study (i.e., [47]) only met six of the nine criteria. With respect to the methodological quality, in three cases both the informed consent was not obtained (i.e., criteria 4 [51, 58, 60]) and the validity of the outcome measures (i.e., criteria 6 [46, 47, 52]) were failed. With only a few exceptions (i.e., criteria 2: detailed description of the sample, and criteria 5: reliable measurement of the outcome both in [47]), all the other criteria were fulfilled in the 16 studies.

### Objective 2: Empirical evidence of prognostic relevance of psychological predictors in soccer

The psycholgocial factor which had the greatest representation was *technical skill* with ten studies, while four studies explored the prognostice relevance of *perceptual-cognitive factors*. Eight studies examined *personality-related dispositions and/or mental skills*.

#### Psychomotor factors

Table 2 provides an overview of the ten studies that examined the prognostic relevance of technical skills. The predictor dribbling (*n* = 9) was the most investigated skill, with seven out of nine studies finding at least one significantly positive relationship with future performance level, indicating its prognostic relevance. The reported or reconstructed effect ranged from 0.47 ≤ *d* ≤ 1.24. The largest effect sizes were reported by Figueiredo et al. [46] who compared elite players vs. club or drop out players at the talent stage U12-U15 (*d* = 1.24). On the other hand, Deprez, Fransen, Lenoir, Philippaerts, and Vaeyens [54], who investigated different development stages, found the smallest yet still moderate effect size (*d* = 0.47) between club and drop-out players. The results of Gravina et al. [47] did not reveal significant differences in dribbling skill when comparing adolescent first team regular players and reserve players based on coaches’ ratings, and this result was also supported by Zibung, Zuber, and Conzelmann [57] when considering the predictor dribbling individually. For ball control (*n* = 6), in five out of six studies, players who performed significantly better, went on to have future soccer success. The reported effect sizes were moderate to large (0.57 ≤ *d* ≤ 1.28). Shooting (*n* = 2) and juggling (*n* = 2) received less consideration. Only Höner and Votteler found [50] a small to moderate significant effect (*d* = 0.28), with youth national players outperforming non-selected players in a shooting test. Finally, Zuber, Zibung, and Conzelmann [53] chose a holistic concept for investigations about talent research based on a person-oriented approach. This study with youth soccer players who were members of regional teams of the Swiss Football Association revealed that highly-skilled players with above average performances in a technical score consisting of dribbling, ball control and juggling skills, might be assumed to receive a higher-than-random number of future youth national players (*d* = 1.04).

**Table 2:** Overview about studies investigated psychomotor factors and their empirical evidence.

Objective 1										Objective 2	
* *	Participants			Measurement Methods			Design and Statistical Analysis			Empirical Evidence	
*Study*	*Size (N)*	*Development stage*	*Performance level T1*	*Investigated predictor variable*	*Diagnostic*	*Criterion variable (Selection level T2)*	*Prognostic period*	*Dimension of domains*	*Procedures*	*Significant predictor variable*	*Significant effect sizes between groups (reconstructed if not given)*
Deprez et al. (2015)	388	<U12, U12-U15, U16-U19	youth academies Belgium	dribbling skill	UGent dribbling test	continue playing level (club vs. drop-out)	2 years	unidimensional	MANOVA, t-test	dribbling skill	d = 0.47* (< U12)
Figueiredo et al. (2009)	159	U12-U15	regional clubs Portugal	dribbling skill, ball control, shooting accuracy, passing skill	test battery of the Portuguese Football Association	continue playing level (elite vs. club and drop-out)	2 years	multidimensional	MANOVA, ANOVA, post-hoc	dribbling skill	d = 1.24*
Forsman et al. (2016)	114	U16-U19	youth academies Finland	dribbling skill, ball control	dribbling and passing test, passing and centering test	professional (elite vs. sub-elite)	4 years	multidimensional	logistic regression, t-test	dribbling skill, ball control	d = 0.61*, d = 0.84*
Gravina et al. (2008)	66	<U12, U12-U15	talent development program Spain	dribbling skill	slalom dribble test	coaches rated the performance of their players (FTP vs. R)	< 1 year	unidimensional	ANOVA; t-test	none	n.s.
Höner & Votteler (2016)	22843	U12-U15	talent development program Germany	dribbling skill, ball control, shooting	motor test battery of the German Football Association	drafted for youth national teams (NT vs. RA and YA and NS)	4–7 years	unidimensional	ANOVA, Tukey's test	dribbling skill, ball control, shooting	d = 0.61*, d = 0.57*, d = 0.28*
Huijgen et al. (2014)	113	U16-U19	youth academy Netherlands	dribbling skill	SlalomSDT, ShuttleSDT	continue playing level (selected vs. deselected)	< 1 year	multidimensional	MANCOVA, Post-hoc; Discriminant analysis	dribbling skill (only ShuttleSDT)	d = 0.60*
Huijgen et al. (2013)	270	U12-U15, U16-U19	youth academy Netherlands	ball control	Loughborough soccer passing test	continue playing level (selected vs. deselected)	< 1 year	unidimensional	multilevel modelling, t-test	ball control	d = 0.69* (U16-U19)
Huijgen et al. (2009)	131	U12-U15, U16-U19	youth academy Netherlands	dribbling skill	shuttle dribble test	professional (professional vs. amateur)	2–6 years	unidimensional	Multilevel modelling	dribbling skill	d = 0.67* (U12-U15), d = 0.77* (U16-U19)
Zibung et al. (2016)	104	U12-U15	talent development program Swiss	dribbling skill, ball control, juggling	motor test battery of the German Football Association	drafted for youth national team or regional clubs (NT vs. RC and NS)	1 year	unidimensional	LICUR analysis	ball control	d = 1.28*
Zuber et al. (2016)	119	U12-U15	talent development program Swiss	dribbling skill, ball control, juggling	motor test battery of the German Football Association	drafted for youth national team or regional clubs (NT vs. RC and NS)	1 year	multidimensional	LICUR analysis	dribbling skill, ball control, juggling	d = 1.04* (technical skill score)

Note. Criterion variable: First team player (FTP); Reserve Player (R); Youth national team (NT); Regional association (RA); Regional club (RC); Youth academy (YA); Non-selected (NS).

*p < 0.05.

#### Perceptual-cognitive factors

Four studies explored the prognostic relevance of perceptual-cognitive factors (see Table 3). In three of these studies, a self-reported tactical skill test (i.e., TACSIS) was utilized that comprises four subscales (i.e., ‘Knowing about ball actions’, ‘Knowing about others’, ‘Positioning and deciding’ and ‘Acting in changing situations’). All three studies highlighted a significant effect size on one of the four subscales. While Forsman, Blomqvist, Davids, Liukkonen, and Konttinen [52] identified a signifcantly better result for elite players (*d* = 0.50) for the subscale ‘Acting in changing situations’, both Huijgen et al. [48] and Kannekens et al. [20] reported that lower performing players showed descriptevly higher values in this subscale. However, Huijgen et al. [48] and Kannekens et al. [20] found significantly better results for future successful players in ‘Positioning and Deciding’ (0.43 ≤ *d* ≤ 0.63). In regard to the video-based assessment procedure used by O’ Connor, Larkin and Williams [56] that included four different tasks (i.e., decision-making, anticipation, pattern recognition and situational probaility), only the decision-making activity significantly discriminated between selected and non-selected players, with a large effect size (*d* = 0.81). When considering descriptive statistics, superior results for selected players were found in anticipation and situational probability but not for pattern recognition.

**Table 3:** Overview about studies investigated perceptual-cognitive factors and their empirical evidence.

Objective 1										Objective 2	
* *	Participants			Measurement Methods			Design and Statistical Analysis			Empirical Evidence	
*Study*	*Size (N)*	*Development stage*	*Performance level T1*	*Investigated predictor variable*	*Diagnostic*	*Criterion variable (Selection level T2)*	*Prognostic period*	*Dimension of domains*	*Procedures*	*Significant predictor variable*	*Significant effect sizes between groups (reconstructed if not given)*
Forsman et al. (2016)	114	U16-U19	youth academies Finland	positioning and deciding, knowing about ball actions, knowing about others, acting in changing situations	TACSIS	professional (elite vs. sub-elite)	4 years	multidimensional	logistic regression, t-test	acting in changing situations	d = 0.50*
Huijgen et al. (2014)	113	U16-U19	youth academy Netherlands	positioning and deciding, knowing about ball actions, knowing about others, acting in changing situations	TACSIS	continue playing level (selected vs. deselected)	< 1 year	multidimensional	MANCOVA, Post-hoc; Discriminant analysis	positioning and deciding	d = 0.63*
Kannekens et al. (2011)	105	U16-U19	youth academy Netherlands	positioning and deciding, knowing about ball actions, knowing about others, acting in changing situations	TACSIS	professional (professional vs. amateur)	3–5 years	unidimensional	Logistic regression	positioning and deciding	d = 0.43*
O'Connor et al. (2016)	127	U12-U15	talent development programme Australia	decision-making, anticipation, situational probability, pattern recognition	video-based assessment	scholarship for an elite player residential programme (selected vs. deselected)	< 1 year	unidimensional	ANOVA Chi-Square analysis Stepwise discriminant analysis	decision-making	d = 0.81*

Note.

*p < 0.05.

#### Personality-releated factors

Regarding *personality-related factors and/or mental skills* (see Table 4), seven studies addressed personality-related dispositions. In this context, four studies analyzed the prognostic relevance of achievement motive for future success. Whereas in most of these studies (*n* = 3) the achivement component hope for success was (significantly) positively associated with future performance (*d* = 0.27; *d* = 0.74; *r* = .27), two of the four studies demonstrated a (significantly) negative association between fear of failure and future soccer success (*d* = 0.21; *d* = 0.30). This relationship was not found by Zuber and Conzelmann (correlation between coaches judgement and fear of failure r = -.01; [60]). Zuber et al. [53] revealed a negative relationship in net hope (*d* = -0.42), which is determined by the difference between hope for success and fear of failure. Furthermore, four studies examined motivational orientations (i.e., performance orientations, assessed by TEOSQ: ego or SOQ: competition, win; and mastery orientations, assessed by TEOSQ: task or SOQ: goal). With regard to performance orientations, three of these studies addressed ego orientation, and none of them found significant relationships between this disposition and future success. Two studies analyzed win orientation and its relationship to future success in soccer. Zuber, Zibung, and Conzelmann [58] revealed that talented soccer players with a higher win orientation (*d* = 0.28) were more likely to obtain a higher performance level compared to players with low win orientation. However, Höner and Feichtinger [55] did not find any significant relationship between win orientation and future soccer success. Additionally, the authors considered competition orientation and found a significantly positive relationship between this type of performance orientation and youth players’ future performance level (*d* = 0.26). Regarding mastery orientations, three studies examined task orientation. The study by Höner and Feichtinger [55] revealed a significantly positive relationship between this variable and future success in soccer (*d* = 0.20), whereas the two other studies did not find any significant associations. Both studies that examined goal orientation [55, 58] found (significantly) positive relationships between this type of mastery orientation and future soccer success (*d* = 0.20; *d* = 0.33). Two studies examined further dispositions within the area of achievement motivation. Van Yperen [51] demonstrated the prognostic value of goal commitment for future success in soccer and found significantly higher values in a group of professionals compared to less successful players (*d* = 0.86). Zuber et al. [58] indicated that talented soccer players with superior self-determination were more likely (*d* = 0.81) to get selected to a higher performance level compared to players with lower self-determination. In addition to motivational characteristics, Höner and Feichtinger [55] examined personality-related dispositions and their relationships with youth soccer players’ future performance level. The volitional competency self-optimization (*d* = 0.23), the self-referential cognitions self-efficacy (*d* = 0.19), specific (*d* = 0.30) and general physical self-concept *(d* = 0.22), and the competition anxiety component worry (*d* = 0.20) were significantly related to future success. In contrast, neither the three volitional deficits (self-impediment, lack of initiation, loss of focus), nor the anxiety components (i.e., concentration disruption, somatic anxiety) were found to be prognostically significant.

**Table 4:** Overview about studies investigated personality-related factors and their empirical evidence.

Objective I										Objective 2	
* *	Subjects			Measurement Methods			Design and Statistical Analysis			Empirical Evidence	
*Study*	*Size (N)*	*Development stage*	*Performance level T1*	*Investigated predictor variable*	*Diagnostic*	*Criterion variable (Selection level T2)*	*Prognostic period*	*Dimension of domains*	*Procedures*	*Significant predictor variable*	*Significant effect sizes (reconstructed if not given) between groups*
Figueiredo et al. (2009)	159	U12-U15	regional clubs Portugal	Goal Orientation	TEOSQ	continue playing level (elite vs. club and drop-out)	2 years	multidimensional	MANOVA, ANOVA, post-hoc	none	n.s.
Forsman et al. (2016)	114	U16-U19	youth academies Finland	Mental Skills	PSIS	professional (elite vs. sub-elite)	4 years	multidimensional	logistic regression, t-test	Motivational skills	d = 0.79*
Höner & Feichtinger (2016)	1701 / 1804	U12-U15	talent development program Germany	Achievement Motive, Sport Orientation, Task and Ego Orientation, Volitional Components, Physical Self-Concept, Self-Efficacy, Competition Anxiety	AMS; TEOSQ; SOQ; VCS; PSC; SES; CAI-T	drafted for youth academies (YA vs. NS)	4 years	unidimensional	Logistic regression analysis, ANOVA, Tukey test	Hope for success, fear of failure, completion orientation, goal orientation, task orientation, self-estimation, general self-concept, specific self-concept, self-efficacy, worry	d = 0.27*, d = 0.21*, d = 0.26*, d = 0.20*, d = 0.20*, d = 0.23*, d = 0.22*, d = 0.30, d = 0.19*, d = 0.20*
Huijgen et al. (2014)	113	U16-U19	youth academy Netherlands	Goal Orientation, Mental Skills	TEOSQ, PSIS	continue playing level (selected vs. deselected)	< 1 year	multidimensional	MANCOVA, Post-hoc; Discriminant analysis	none	n.s
Van Yperen (2009)	65	U16-U19†	youth academy Netherlands	Goal Commitment, Coping, Seeking Social Support	Van Yperen Scale	professional (successful vs. unsuccessful)	15 years	unidimensional	ANCOVAs; Discriminant analysis	Goal Commitment, Coping, Seeking Social Support	d = 0.86*, d = 0.6*
Zuber et al. (2016)	119	U12-U15	talent development program Swiss	Achievement Motive	AMS	drafted for youth national team or regional clubs (NT vs. RC and NS)	1 year	multidimensional	LICUR analysis	none	n.s.
Zuber et al. (2015)	92	U12-U15	talent development program Swiss	Achievement Motive, Orientation, Self-Determination	AMS, SOQ	drafted for youth national teams (NT vs. NS)	1 year	unidimensional	LICUR analysis	Hope for success, self-determination	d = 0.74*, d = 0.81*
Zuber & Conzelmann (2014)	140	U12-U15	talent development program Swiss	Achievement Motive	AMS	coaches rated the performance of their players (fictions NT vs. NS)	< 1 year	unidimensional	Structural equation models	Hope for success	r = 0.27*

Note. Criterion variable: Youth national team (NT); Regional club (RC); Youth academy (YA); Non-selected (NS)

*p < 0.05

†There are a few players who are under the age of 16

To address mental skills, two studies used the Psychological Skills Inventory for Sports (PSIS). Forsman et al. [52] found a significantly positive relationship between motivational skills and the future performance level of youth soccer players (*d* = 0.79). In comparison, Huijgen et al. [48] did not find motivational skills to be significant predictors, and these skills were negatively associated with future success. Furthermore, Van Yperen [51] demonstrated that subsequently successful players showed significant higher values in seeking social support (*d* = 0.60).

## Discussion

The aim of this systematic review was to analyze the existing literature regarding the prognostic relevance of psychological talent predictors in soccer. Based on systematic reviews in other talent identification domains (e.g., [36–39]), the number of studies to examine the prognostic relevance of psychological predictors (*n* = 16) seems suitable. However, the large number of different domains of psychological predictors and variable findings across individual factors limits the conclusions that can be drawn. Nevertheless, the findings demonstrated the importance of investigating empirical evidence and considering relevant study design features. In addition to current research, this systematic review provides a detailed analysis of the predictive value of psychomotor, perceptual-cognitive and personality-related factors on soccer performance. Therefore, this article illustrates the key findings of the extant research in relation to psychological characteristics associated with talent development in soccer.

### Relevant design features of existing research on the prognostic value of psychological factors

In order to discuss current trends in the literature, it should be noted that heterogeneous study designs have an impact on the findings and limit the scope of accurate conclusions. Therefore, one aim of the present study was to provide an overview of the methodological approaches of the researchers. With respect to *participants* country of origin, it is noteworthy that psychological predictors are largely ignored in nations such as the United States or the United Kingdom, where comprehensive talent research is predominant [36]. While in the United States other team sports (e.g., American Football or basketball) are more popular [61], explaining the focus of research on those activities, it is surprising that for soccer in the United Kingdom, which is known as the homeland of the game, there are no recent studies investigating the predictive value of psychological predictors. Moreover, there is a dearth of studies investigating prognostic relevance for youth female athletes. These results are in line with other studies that highlighted a lack of research in talent development and identification in female soccer, even as participation and professionalization has recently increased [39, 62].

Referring to developmental stages, there are very few investigations (*n* = 2) with players under 12 years of age. This could be attributable to several factors. For instance, federations often do not start a systematic talent identification and promotion program before the age of 12 (e.g., [48]). Another reason may be the complex and dynamic nature of the development process of youth players and the question about efficacy of early identification in general [63]. Some researchers have questioned the applicability of objective assessments due to the unstable performance development of youth athletes (especially in early developmental phases; [2]). In addition, scholars have argued such diagnostics often consist of test batteries that assess performance independent of athletes’ maturity [64], which results in the frequently discussed relative age effect that can lead to maturation-related biases in diagnostics (e.g., [65, 66]). While fluctuations in physical and physiological characteristics throughout (adolescent) development is well-etablished [67], information about the stability of psychological factors and how they adjust during early years of an athlete’s career are limited [36, 68]. Consequently, for a comprehensive understanding of talent identification and development, it would be worthwhile for researchers to investigate the prognostic relevance of individual talent predictors in all developmental stages [5], which would provide greater insight into the importance of possible structural adjustments for a given factor in a particular stage of factors.

To compare study results from different research groups it is necessary to correctly classify the investigated participation performance level at T1. In one study Forsman et al. [52] noted the different findings of comparing the performance level of a youth academy player in Finland with such players in the Netherlands or Germany. Except for Figueiredo et al. [46], all studies included in this review explored predictors for players attending a youth development program or who were members of youth academies, thus representing a high performance level group. This result aligns with Toering et al. [35] who corroborate more comparisons within elite groups. When reporting participant levels, only a few studies provide readers with an exact description of the performance level of players. For instance, Huijgen et al. [1] reported that the investigated players belong to the best 0.5% of the total number of Dutch soccer palyers in their age group. For future research it would be helpful to present more detailed information for instance, a percentage value for the performance level of the meausered players (e.g., best 1% players at the age group U15).

Similar discrepancies are present in the *measurement methods* relating to the terminology used by researchers to express the selection level at the subsequent measurements T2. The substantial variation in largely self-determined definitions by the authors (e.g., selected for next age group, achieving a professional contract, drafted for youth national team) impede comparability between the studies. This observation is in line with previous research by Swann, Moran and Piggott [69] and Johnston et al. [36], who highlighted an inconsistency in the terminology of skill levels. Frequently, the comparison of perfomance levels between different professional leagues is a challenge, as is the way authors define future success for players. Vaeyens et al. [22] stressed that the main aim of talent development programs is to identify young athletes with the potential for elite success in adulthood. Nevertheless, only four of the included studies in this review chose this selection criteria. In the future, researchers can maintain current approaches, and, where possible, also follow the players into adulthood. Furthermore, it appears desirable to use more consistent measurements to adequately compare study results and make meaningful conclusions about the prognostic relevance of certain predictors. Understandably, the large domain of psychological predictors requires the implementation of specific types of diagnostics. In this context, psychometric properties of measurement instruments are crucial for investigating talent predictors and increase the comparability of results [70]. Another option to compliment classical testing that would provide relevant insights into performance would be to use an inventory of instruments in which players self-report their performance in combination with an external judgement by experts. For example, Musculus and Lobinger [71] provided recommendations on how to ensure scientifically sound coaches’ assessment of psychological characteristics.

With regard to *design and statistical analyses*, large sample sizes such as Höner and Votteler [50] constituted an exception. This finding emphasizes the appeal by Mann et al. [3] for more large-scale longitudinal studies. Vaeyens et al. [22] also suggested that the length of the prognostic period influences the effects associated with the individual factors’ predictive relevance. The majority of the studies in this review investigated prognostic relevance over a short or middle term period (three years or less). On the one hand, consideration of shorter periods can help to understand important transitions in adolescence, but can also be more susceptible to confounding factors such as instability. For instance, very short prognostic periods in adolescence could be detrimental to late matured players because of physical handicaps (e.g., height and weight). However, the more relevant question for talent development programs should be which factor indicate that an athlete has the potential to develop positively and become a successful player in adulthood [22]. Even better would be studies that combine investigations of developmental processes and the predictive value in different age groups [72]. Therefore, long-term prognostic periods are of interest and have the most practical merit. Due to the complex spectrum of talent predictors Till and colleagues [73] preferred to adapt a multidimensional approach as was conducted by Forsman et al. [52] or Huijgen et al. [48]. In contrast to Tills’ et al. [73] perspective, the majority of authors investigated unidimensional approaches which has been criticized in previous research [22, 74]. In a statistical context, a multidimensional approach provides the possibility of using both univariate und multivariate analysis. Therefore, on the one hand a crucial point is an individual consideration of the factors (e.g., prognostic relevance of individual factors), and on the other hand multidimensional diagnostics or procedures are important as part of complex theory models. For instance, some of the studies used a multidimensional design and applied multivariate statistics. Whenever this was conducted, no significant effects of personality-related characteristics were revealed. This may be explained by the fact that psychological dispositions and skills–compared to, for example, technical skills–only explain a small portion of future performance, and therefore their influence gets lost in multidimensional, multivariate designs. An exception to this observation is the study by Forsman et al. [52] which revealed a significant effect of motivational skills. However, this study showed contradictory results in comparison with other research [48]. For a deeper discussion of different analytical procedures see Höner et al. [12].

To sum up, central features of study design may influence the prognostic relevance of individual talent predictors. Based on the heterogeneous methodological approaches, the ability to report accurate conclusions regarding prognostic relevance is limited. For instance, independent of the developmental stage, significant effects were found for various predictors. From a practitioners’ perspective it would be more valuable to specify important factors for different stages.

In previous systematic reviews, the *methodological quality of eligible studies* has been evaluated using well-established assessment tools (e.g. PEDROscale, Mixed-model appraisal tool, MINORS [75–77]). However, most of these evaluation scales were designed for intervention studies and not applicable to the papers within the current review. Using an adapted version of the Critical Review Form for quantitative studies to compare the methodological quality of the empirical literature on male soccer talent identification and development [44], the studies included in this current review nearly fulfilled all chosen quality criteria, while only a few studies did not meet the quality assessment (e.g., criteria 4; informed consent was not obtained). This is in line with Sarmentos’ review in which the average fulfillment of criteria for 63 selected quantitative studies is very high, too.

Overall, there seems to be minimal benefit in applying existing quality assessment tools, which are generally used for intervention studies, to the talent identification and development research. Therefore, future efforts should be directed at defining methodological quality assessment criteria which are 1) described in detail, and 2) relevant to the talent identification and development literature. Based on the analysis of the respective study design features (objective 1) this review provides an opportunity for identifying appropriate assessment criteria. For instance, with respect to participants, it is not sufficient to state general descriptors (e.g., number of participants, age, country etc.), but rather, more detailed information should be provided, such as levels of performance (e.g., detail categorization within the investigated country; elite, sub-elite, novice). Further, more comprehensive assessment of the statistical analyses undertaken would improve the interpretation of results (e.g., in addition to descriptive statistic, both uni- and multivariate examination in investigating multidimensional predictors). With respect to measurement methods, more specific criteria concerning the psychometric properties of diagnostics (e.g., determination of instrumental reliability and criterion-related validity) would support the methodological quality of studies. Therefore, future studies in the talent identification and development area, should consider the development of a methodological quality assessment measure which considers some of these potential criteria. In doing so, this would more appropriately assess the strength and quality of the talent identification and development studies, compared to more well-established tools (e.g. PEDROscale, Mixed-model appraisal tool, MINOR).

### Empirical evidence of prognostic relevance of psychological predictors in soccer

Analyzing the number of studies dealing with psychological predictors revealed an imbalance between more frequently regarded technical skills and personality-related factors on the one side and relatively underrepresented perceptual-cognitive factors on the other side.

The fact that most studies investigate the prognostic relevance of dribbling and ball control could be based on previous literature in which studies demonstrated the importance of these key factors (e.g., [78]). The results of this review support previous findings that emphasize the importance of both technical skills independent of the investigated development stage. In almost all reviewed studies, the prognostic relevance of dribbling and ball control (e.g., passing or trap the ball) was significant with moderate to large effect sizes (0.47 ≤ *d* ≤ 1.28). By contrast, only two authors investigated the prognostic relevance of the factor shootin*g*, despite its central role in scoring goals in games. At this juncture, it appears surprising that a factor as essential as shooting only revealed a low predictive value. The gap in the literature with regard to shooting could be a result of the complexity of this characteristic and the difficulty of developing reliable measurements [79, 80]. One possible approach to receive more attention for shooting is the development of standardized shooting tests (at least for certain playing positions (e.g., forwards)), in combination with subjective judgements from expert coaches. In the current research, the factor juggling also received limited consideration. This finding is likely due to juggling being an activity, conducted in training or leisure time, not a key component of in-game performance.

A possible reason for the lack of studies about the predictive value of perceptual-cognitive factors might be the difficulty in capturing such latent variables. Appropriate diagnostic instruments are often very time-consuming and complex, especially in a sport-specific context. In this review, three studies examined the predictive value of perceptual-cognitive factors using TACSIS and highlighting contradictory significant effect sizes between higher and lower performing players. However, the use of self-reported tactical skills to examine perceptual-cognitive skills should be considered critically. In this context, Nortje, Dicks, Coopoo and Savelsbergh, (see p. 330 in [81]) argued that there is a difference between self-reported questionnaires and real game situations where players are “competing against opposing players and cooperating with their teammates”. With regard to the prognostic relevance of decision-making, O’ Connor et al. [56] demonstrated a large significant effect size (d = 0.81) utilizing a soccer-specific video-based assessment. Overall, the results indicated a lack of studies examining perceptual-cognitive skills with a perceptual-action coupling which would be closer to real game situations.

With respect to personality-related factors, most of the research examined the prognostic relevance of psychological dispositions, and only a few studies considered mental skills. In the context of talent research, this may make sense, because dispositions are regarded to be more stable over time and across situations than state-based skills, which can change from situation to situation or from day to day [82]. A number of studies demonstrated that psychological dispositions and skills discriminate between youth players of different performance levels (e.g., [51, 55, 58]). However, other research has found no significant differences between high- and low-performing youth players with regard to such characteristics (e.g., [46, 48]). Furthermore, some of these studies have reported contradictory results (e.g., [48, 52]). Within the motivational characteristics, both components of the achievement motive (i.e., hope for success and fear of failure) assessed by the AMS-S seem to be associated with future success in soccer. In the majority of studies, hope for success was positively associated with future performance and fear of failure was negatively related to success. These findings support previous research (for an overview, see [83]) that revealed athletes with high dispositions toward hope for success demonstrated more functional behaviors (e.g., more endurance and effort, and self-serving attributions) compared with the individuals with high fear of failure values. Regarding the prognostic relevance of motivational orientations, sport psychology talent research provides findings that are more heterogeneous. Out of three studies addressing the prognostic relevance of goal orientations (i.e., ego and task orientation assessed by the TEOSQ), only Höner and Feichtinger [55] found a significant relationship between task orientation and future success. In comparison to the TEOSQ, the SOQ seems to be a more reliable assessment of motivational orientations in the context of sport talent research. Although Höner and Feichtinger [55] did not find win orientation to be a significant predictor of future success, their results revealed relevant associations between the SOQ subscales competition, goal and win orientation and players’ future performance. As a consequence, further studies examining motivational orientations in sport talent research might prefer the SOQ over the TEOSQ because both questionnaires have the same theoretical foundation [84]. A small number of studies examined further motivational dispositions (e.g., goal commitment, self-determination) or characteristics from other personality domains such as volition, (self-referential) cognition, and emotion. This limited research can only provide an initial understanding of the prognostic relevance of personality-related dispositions such as volitional competencies, self-concept, self-efficacy, and competition anxiety. The same applies for mental skills. Nevertheless, mental skills play an important role in athletic performance [85], and more prognostic studies are needed to be able to make reliable statements about their relevance for future success. In the area of personality-related dispositions, the focus so far has been on motivational characteristics (i.e., achievement motive, motivational orientations). For other personality areas, there are only limited prognostic studies (e.g., [55]), which only provide an initial exploration of the relevance of volitional, (self-referential) cognitive, and emotional dispositions. Given the inconsistent state of empirical research (e.g., with regard to motivational orientations or mental skills), the relationship between personality-related characteristics and future performance level in soccer requires further examination. It should be mentioned (again) that different design features of the studies may have influenced the inconsistencies of all considered factors in this review.

## Conclusion

The current study provided insights into the prognostic relevance of psychological talent predictors for young soccer players and complemented the review of the predictive value of physical and physiological characteristics by Murr et al. [39]. Evidence was found for individual factors (e.g., dribbling, decision-making, achievement motive), however, additional research is warranted for investigating individual talent predictors more comprehensively. Large-scale studies that employ multidisciplinary test batteries to assess youth athletes at different age groups are required to improve the specificity of predictions [3]. Such approaches can provide clubs and coaches with valuable information to support the promotion of talented players within their organizations. Moreover, the prognostic relevance of personal talent predictors relating to different playing positions might be of future interest. Besides person-oriented factors, environmental factors (e.g., training or game play activities that may influence the level of ability a player can attain) should be considered as well [86].

## Supporting information

S1 Table PRISMA checklist. From: Moher D, Liberati A, Tetzlaff J, Altman DG, The PRISMA Group (2009). Preferred Reporting Items for Systematic Reviews and Meta-Analyses: The PRISMA Statement. PLoS Med 6(7): e1000097. doi:10.1371/journal.pmed1000097.
(DOCX)

S2 Table Criteria used to analyse the methodological quality of studies (adapted from Sarmento et al., [44]). Note. * (if not described, assume No).
(DOCX)
