# Supplementary material for: Psychological talent predictors in youth soccer: A systematic review of the prognostic relevance of psychomotor, perceptual-cognitive and personality-related factors
Source: PLoS One. 2018 Oct 15;13(10):e0205337. doi: 10.1371/journal.pone.0205337 (PMC6188900; doi:10.1371/journal.pone.0205337)
Supplement: S2 Table — Note. * (if not described, assume No). (DOCX) [file pone.0205337.s002.docx]

**S2 Table.** - Criteria used to analyse the methodological quality of studies (adapted from Sarmento et al., [44])

Note. * (if not described, assume No)
